# Formation of Conductive DNA-Based Nanowires via Conjugation of dsDNA with Cationic Peptide

**DOI:** 10.3390/nano7060128

**Published:** 2017-05-30

**Authors:** Zeinab Esmail Nazari, Julio Gomez Herrero, Peter Fojan, Leonid Gurevich

**Affiliations:** 1 Institute of Physics and Nanotechnology, Aalborg University, DK-9220 Aalborg, Denmark; esmailnazari82@gmail.com (Z.E.N.); fp@nano.aau.dk (P.F.); 2 Department de Fisica de la Materia Condensada, Universidad Autonoma de Madrid, 28049 Madrid, Spain; julio.gomez@uam.es

**Keywords:** DNA nanotechnology, electrostatic force microscopy (EFM), DNA conductivity, DNA-peptide conjugates, self-assembly, surfactant peptides

## Abstract

A novel conductive DNA-based nanomaterial, DNA-peptide wire, composed of a DNA core and a peripheral peptide layer, is presented. The electrical conductivity of the wire is found to be at least three orders in magnitude higher than that of native double-stranded DNA (dsDNA). High conductivity of the wires along with a better resistance to mechanical deformations caused by interactions between the substrate and electrode surface make them appealing for a wide variety of nanoelectronic and biosensor applications.

## 1. Introduction

During the last two decades, DNA has become one of the cornerstones of nanotechnology. Its self-assembling properties, robustness, and well-developed enzymatic machinery have led to the design of intricate nanodevices, ranging from DNA arrays to nanorobots (see for review [1]). On the other hand, the presence of nearly parallel stacked aromatic groups in dsDNA with a distance similar to that between graphite layers has also placed DNA in the focal spot for molecular electronics and led to a vast array of different studies with often controversial results (for a thorough discussion we address the reader to reviews [2,3]). Although the understanding of both experimental and theoretical aspects of DNA conductivity is far from complete, the growing body of evidence suggests that short dsDNA molecules (<40 nm) generally exhibit semiconductor-like behavior with a gap in the range of ~1–2 V [4,5]. This was first reported by Porath et al. [4], who studied electrical transport through relatively short (10 nm) dsDNA molecules deposited between platinum nanoelectrodes at different temperatures. This type of behavior was later observed in several other studies, confirming reproducible semiconducting behavior with a gap [5,6]. Although both hole and electron transport are possible in DNA according to photophysical studies [7], electrical studies indicate that the charge transport is dominated by holes, presumably due to the positioning of the HOMO (Highest Occupied Molecular Orbital) and LUMO (Lowest Unoccupied Molecular Orbital) levels of DNA with respect to the Fermi energy of common metal contacts (e.g., Au and Pt). As a result, a DNA molecule behaves as a *p*-type nanowire in electrical measurements [8]. The charge transport in DNA occurs predominantly through guanine bases due to their lowest electrochemical oxidation potential. Three main mechanisms have been proposed to explain charge transport in DNA molecules: single-step-electron-tunneling, thermal hopping, and domain hopping [9].

The conductivity of long (hundreds of nanometers) DNA strands appears to be the most complicated part of this puzzle. Once a DNA molecule is adsorbed on a solid surface, its conformation is perturbed due to van der Waals, electrostatic, and hydrophobic interactions with the substrate. The behavior of long DNA molecules is thus affected by a number of experimental parameters such as DNA sequence, substrate and contact properties, temperature, and humidity [10,11,12,13]. Since the charge transport properties of DNA critically depend on π-orbital stacking along the molecule [9], even local perturbations of the double-stranded structure could lead to a dramatic reduction of the ability of DNA to conduct electrical current. This “fragility” of charge transport through DNA is also supported by electrochemical measurements in solution [14]. While observed currents through well-matched 17-mer and 100-mer dsDNA monolayers are rather similar, even a single mismatch breaking π-stacking at one point is sufficient to reduce the current by half. One of the ways to overcome this issue is by forming more rigid DNA structures that are less prone to surface-induced deformations. Recently, Livshits et al. [15] performed electrical measurements on guanine quadruplex DNA (G4-DNA) which is uniform in composition consisting of only G-nucleotides and exhibits a greater bending rigidity compared to dsDNA due to the guanine tetrad as a repeating unit. Although these molecules have been found to support long range charge transfer at distances approaching 100 nm, the conductive behavior of the molecules is described as variable-range hopping. This indicates that even such a rigid DNA structure experiences significant perturbation when deposited on a solid substrate, which leads to the reduction of electron delocalization down to a few tetrads. A different approach can involve the reduction of the DNA-surface interaction through, e.g., introducing electrostatic repulsion between the DNA and the surface [13], which also leads to a certain improvement in dsDNA conductivity.

In the present study, we addressed another route to stabilize the native structure of DNA on a surface by coating it with a layer of cationic molecules, specifically, cationic peptides. Short cationic peptides have recently attracted significant interest due to their antimicrobial properties and ability to form very stable self-assembled structures with or without templates [16,17,18]. Some of them have been proven to form stable uniform coatings around dsDNA [19].

In the present study, we investigated the conductivity of DNA-peptide nanowires both contactless (using Electric Force Microscopy (EFM)) and through direct I–V measurements with nano-fabricated contacts. The results clearly demonstrate that coating with KA_6_, a cationic peptide composed of one lysine and six alanine residues, leads to almost a thousand-fold increase in conductivity of the DNA.

Metallic Carbon Nano Tubes (CNTs) were used as a fiducial reference 1D conductor during all procedures, since the electrical properties of this material has been well studied using both EFM and I–V measurements [20,21].

## 2. Results and Discussion

**Figure 1:**
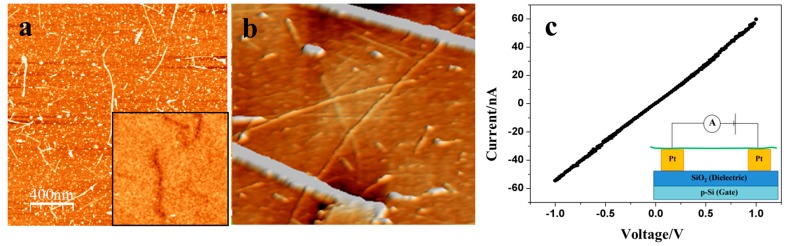
(**a**) Topography image of metallic carbon nanotubes (CNT) deposited on silicon wafer obtained using an atomic force microscope (AFM) operating in the intermittent contact mode. The insert shows a phase image of the same sample acquired using two-pass electrostatic force microscopy (EFM) at a tip bias of +3 V and a lift distance of 20 nm. CNTs are visible as white lines in the topography image due to their height, and as black shadows in the EFM phase image due to their conductivity; (**b**) AFM topography image (3D representation) of CNTs deposited across two nanoelectrodes; (**c**) The corresponding I–V curve obtained with a voltage sweep from −1 V to +1 V with voltage steps of 0.1 V. The insert shows a schematic representation of the I–V measurement setup. The electrodes were bridged by several CNTs at distances ranging from 60 to 600 nm.

The results of the reference experiments performed using metallic CNTs for both EFM and I–V measurements are illustrated in Figure 1. In line with the previous studies [20,22,23], CNTs displayed a clear phase-shift signal in the EFM phase images (Figure 1a, inset), often referred to as EFM polarizability, and produced linear I–V curves indicating that functionalization with silanes does not significantly affect the contact properties of Pt electrodes.

**Figure 2:**
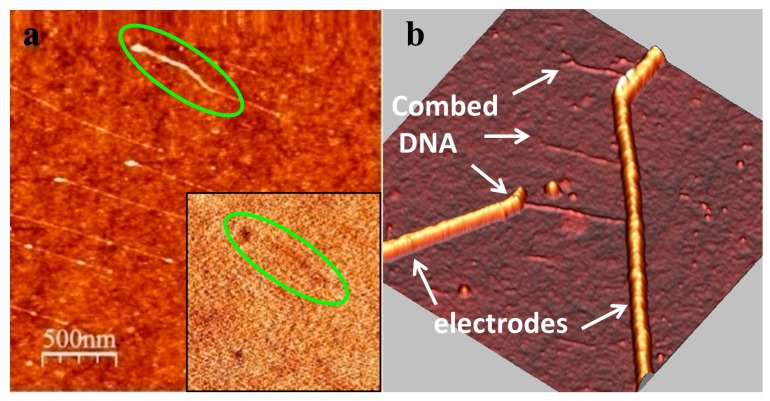
(**a**) AFM topography of dsDNA molecules combed on a silicon substrate. The inset shows a very poor phase contrast obtained in EFM at +8 V bias and 10 nm lift height; (**b**) AFM image of dsDNA combed across nanoelectrodes.

Under the same experimental conditions, dsDNA stretched on the substrate exhibited a very poor electrostatic contrast, even when the tip bias voltage in EFM experiments was increased to ±8 V and the lift distance was lowered down to 10 nm (Figure 2a). The contrast quickly disappeared at higher lift distances of 20 and 30 nm (data not shown), in line with the previous studies [22]. Consistent with the results obtained by Maragakis et al. [24] and Heim et al. [25] on the absence of conductivity in over-stretched DNA, in our experiment the signal was completely absent for over-stretched DNA molecules, most probably due to drastic changes in the conformation and induced perturbation in π-stacking. In agreement with the EFM measurements, the I-V curves obtained for dsDNA molecules combed across two adjacent nanoelectrodes (Figure 2b) indicated currents within the noise level (~50 fA).

**Figure 3:**
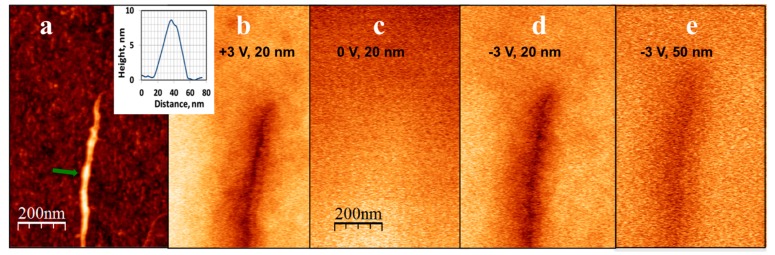
(**a**) Topography of a DNA-KA_6_ conjugate combed on a silicon surface. The inset shows the height profile of the conjugate (the arrow in the main panel marks the position of the cross-section). Corresponding phase images of the same feature obtained in EFM mode at: (**b**) +3 V bias voltage and a lift distance of 20 nm; (**c**) 0 V and a lift distance of 20 nm (**d**) −3 V and a lift distance of 20 nm; and (**e**) +3 V and a lift distance of 50 nm. The phase-shift signal (the dark region around the conjugate location), which broadens as the lift distance increases and vanishes when the tip bias voltage goes to zero, is clearly visible. The scale is the same in all panels.

Interestingly, both the EFM polarizability and I–V behavior of DNA were drastically changed when DNA was conjugated with KA_6_ peptides. DNA-KA_6_ conjugates deposited and combed on functionalized silicon substrates produced a strong phase shift in EFM measurements at the same parameters at which dsDNA exhibited no signal. Figure 3b–e shows the results of EFM measurements on a DNA-KA_6_ conjugate. The phase shift due to electrostatic interactions between the metal-coated tip and the conjugate is visible as a dark shadow around the conjugate. It can be seen that the contrast vanishes at zero voltage between the tip and the substrate and gradually broadens and disappears as the tip is removed from the surface (as the lift height increases). The phase-shift signal was still detectable at distances as high as 100 nm, suggesting significantly enhanced conductivity of the DNA-peptide wire with respect to the non-coated DNA.

**Figure 4:**
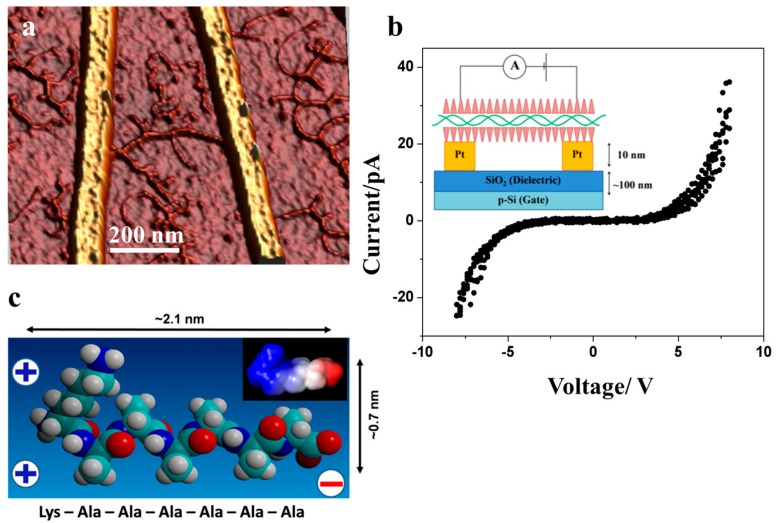
(**a**) AFM topography image (3D representation) of DNA-KA_6_ conjugate deposited and combed across two adjacent electrodes; (**b**) The corresponding I–V curve obtained with voltage sweeps of −8 V to +8 V with voltage steps of 0.1 V, three consecutive sweeps overlaid. The inset shows the schematics of the experiment. The electrodes were bridged by a single conjugate of ca. 300 nm; (**c**) Space-filling model of KA_6_, a surfactant-like peptide with charge separation. At moderate pH values (pH 4–8) the peptide possesses two positive charges at one end (N-terminus and lysine) and a negative charge at the opposite end (C-terminus). The inset shows an electrostatic map indicating the charge distribution at pH = 7 [18]. The electrostatic map was calculated using TITRA [26] and mapped onto the molecular surface of the peptide with DELPHI [27].

Figure 4a demonstrates the deposition and combing of DNA-KA_6_ conjugates across nano-fabricated electrodes used for I–V tests. In agreement with our EFM results, the I–V characteristics measured on DNA-KA_6_ conjugates showed a significant improvement compared to the native form of DNA. The I–V curves for the DNA-peptide wire were nonlinear with a fairly symmetric gap from approximately −5 to +5 V (it should be noted that the actual voltage applied to the conjugate is lower due to the contact resistance). We performed measurements on two different devices, both of which showed a qualitatively similar behavior with a similar gap and activation energy values. Our consecutive atomic force microscopy (AFM) measurements on one of the devices indicated the length of the DNA-peptide wire to be about 300 nm. The current produced after sweeping the voltage up to 8 V was almost three orders in magnitude higher for DNA-peptide wires (~40 pA) than for native DNA (~50 fA). This seems to be a logical extension of the established results on short DNA strands, which reported nonlinear I–V characteristics with a voltage gap [4,5], and are qualitatively in line with the conductivity data obtained on extended DNA structures reported earlier [6,10,13,15].

**Figure 5:**
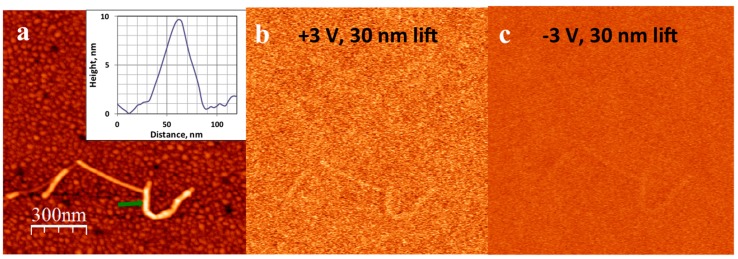
(**a**) Topography of a DNA-KA_5_ conjugate combed on a silicon surface. The inset shows the height profile of the conjugate (the arrow in the main panel marks the position of the cross-section). Corresponding phase images of the same feature obtained in EFM mode at: (**b**) +3 V bias voltage and a lift distance of 30 nm and (**c**) −3 V and a lift distance of 30 nm. No phase shift signal corresponding to EFM polarization (dark shadow) is observed. The scale is the same in all panels.

It should be noted that cationic peptides, due to their positive charge, have a tendency to induce the aggregation of DNA. As can be seen in Figure 3 and Figure 4, the KA_6_-DNA conjugates commonly form ropes, up to 10 nm in diameter, containing several DNA molecules. A similar tendency and similar size of DNA ropes were observed with other short cationic peptide used in the study: IL, IL4, KA_5_, and KA_6_W. However, conjugation with these peptides did not lead to enhanced electrical properties and the conjugates showed no EFM contrast and I–V characteristics similar to that of bare DNA molecules. Figure 5 presents EFM and AFM measurements on a KA_5_-DNA conjugate. The size of the horseshoe structure is very similar to that in Figure 3, however, no EFM polarizability was observed; only a small signal due to the van der Waals forces was visible.

We attribute the enhanced EFM contrast and higher conductivity of the DNA-KA_6_ conjugates to a stabilizing effect of the cationic peptide on the surface-bound DNA molecule. It can be expected that the peptide coat reduces the interaction with the surface and therefore helps to stabilize the native (solution) conformation of the DNA. Furthermore, the conjugated peptides can contribute to maintaining the proper hydration of DNA molecules. As has been shown, both theoretically [28,29] and experimentally [11,12,13], the hydration degree of a DNA molecule strongly (up to one thousand-fold) affects its conductivity. Although the nature of this phenomenon is highly debated, it has been shown that dehydration of DNA significantly increases the band gap of a DNA molecule from ~3.0 to ~8.0 eV [28], and moreover destabilizes the DNA structure, creating structural irregularities [29]. Interestingly, condensation of DNA into ropes by positively charged spermidine has been observed to drastically increase the spreading of the injected charge compared to bare DNA fibers of similar dimensions [25]. On the other hand, small bundles of bare DNA (up to ca. 10 molecules) deposited on a solid surface showed no conductivity under similar measurement conditions [10].

The general assumption is that the cationic peptides are attracted to the negatively charged backbone of DNA and can cover DNA by (partially) replacing the counter ions [16]. We have earlier observed the formation of a coating layer around dsDNA using IL and IL4 peptides [19]. The molecular modeling for the peptide interaction with the DNA template suggests that although the electrostatic interactions drive the peptide molecules towards the DNA, the hydrophobic interactions as well as the actual shape of the peptide are also critically important for peptide docking onto a DNA molecule. For instance, aromatic side chains present in IL and IL4 peptides can significantly alter the structure of the helix, possibly leading to a reduction of the DNA conductivity. Further experimental and computational studies are required to elucidate the interaction mechanism and geometry of DNA-KA_6_ conjugates and explain why KA_6_ produced a remarkable effect on DNA conductivity, while other peptides had no effect at all.

## 3. Materials and Methods

The experiments were performed on a linearized dsDNA plasmid, 2686 base pairs long (pUC19 DNA plasmid/*Sma*I digest, 25 ng/µL, Fermentas Life Sciences, Vilnius, Lithuania). The DNA solution was buffer exchanged to 20 mM ammonium acetate (Sigma Aldrich, St. Louis, MO, USA) solution. IL, IL4, KA_5_, KA_6_, and KA_6_W peptides were produced using automated solid phase peptide synthesis (Activo-P11, Activotec, Cambridge, UK) and were HPLC-purified before use.

Platinum nanoelectrodes were fabricated using a combination of optical and e-beam lithography followed by the evaporation of 1 nm of Cr and 5–10 nm of Pt in a Cryofox Explorer 600 (Polyteknik A/S, Oestevraa, Denmark) and subsequent lift-off. In this way, we could achieve thin continuous Pt/Cr electrodes (width of 30–40 nm, electrode spacing down to 40 nm). The silicon substrate (NOVA Wafers, Flower Mound, TX, USA) on which the nanoelectrodes were fabricated consisted of a layer of thermally grown SiO_2_ (100 nm thick) on top of highly *p*-doped silicon that could also be used as a back gate. Silicon substrates, both plain and bearing nanoelectrodes, were rinsed with acetone followed by UV-ozone cleaning (Bioforce Nanosciences, Salt Lake City, UT, USA) and were functionalized by vapor phase deposition of *N*-Octyldimethylchlorosilane (Sigma Aldrich, St. Louis, MO, USA) for 2 h in a sealed evacuated chamber. The advancing and receding angles of the substrates were measured using the sessile droplet method, yielding an average contact angle of ~90°. The substrates for CNT measurements were treated in the same way but using (3-Aminopropyl) trimethoxysilane (APTMS) (Sigma Aldrich, St. Louis, MO, USA) during the vapor deposition step.

A droplet of DNA or DNA-peptide solution in ammonium acetate buffer (20 mM, pH = 5.1) was deposited on silanized substrates, incubated (6 min) at room temperature and then combed using a variation of the combing technique recently reported by us [30]. Generally, the combing techniques rely on a partial melting of DNA ends under certain conditions and their attachment to solid substrates (see [31] for a review). Then, the DNA molecules can be stretched and aligned on the substrate by applying, e.g., a meniscus force in a given direction.

The stock peptide solutions of KA_5_ (8 mM), KA_6_ (4 mM), and KA_6_W (4 mM) in ammonium acetate (20 mM) were sonicated (30 min) prior to mixing with DNA. IL (680 µM) and IL4 (20 µM) were used without sonication. The DNA solution was mixed in a ratio of 1:15 with the stock solution of peptide in question and incubated for 2 h. Interestingly, our variation of the combing method proved to be efficient in combing both dsDNA and DNA-peptide conjugates, while other commonly used combing recipes were ineffective for the combing of DNA-peptide conjugates.

Predominantly metallic single-wall carbon nanotubes were obtained from Carbon Nanotechnologies, Inc., Houston, TX, USA. De-bundling of CNTs was achieved by sonication in 1.5% sodium cholate solution (Sigma Aldrich, St. Louis, MO, USA). Prior to the deposition, the silicon substrates were treated with APTMS vapor for 1 h as described above.

AFM and EFM measurements were carried out using a Multimode AFM with Nanoscope IIIa controller (Bruker, Billerica, MA, USA), operating in tapping and lift (two-pass) modes, respectively. OMCL-AC200TS, OMCL-AC240TS (Olympus, Tokyo, Japan), and HR-SCC (Team Nanotec GmbH, Villingen-Schwenningen, Germany) cantilevers were used for AFM imaging. For EFM studies, conductive cantilevers were prepared by evaporating layers of 1 nm Cr and 5 nm Pt on OMCL-AC240TS cantilevers. The EFM measurements followed the method for contactless evaluation of conductivity developed in [20,22]. AFM and EFM images were processed using the WSxM software package [32]. I–V characteristics were acquired with a Model 6517A (Keithley Instruments Inc., Cleveland, OH, USA) source-electrometer using a custom LabView (National Instrument, Austin, TX, USA) program. The measurements were carried out in a two-point geometry using a PM5 probe station (SÜSS MicroTec, Garching bei München, Germany) placed inside a Faraday cage, leading to a typical noise level below 150 fA rms. To exclude a possible leakage through the oxide layer to the silicon back gate, additional I–V measurements between bare electrodes as well as the electrodes and the back gate were carried out on all measured devices. Data from several voltage sweeps were acquired to ensure that the curves are reproducible. A voltage up to ±10 V applied to the back gate did not produce any current leakage within the experimental accuracy. The measurement equipment was located in an ISO Class 5 clean room with a continuous control of temperature and humidity, set to 21 ± 1 °C and 45 ± 5% RH, respectively.

## 4. Conclusions

We demonstrated that the binding of a cationic peptide, KA_6_, leads to a dramatic increase in the conductivity of dsDNA, measured both contactless using EFM and by direct I–V measurements on molecules placed on nanoelectrodes. The observed EFM phase shift above DNA-KA_6_ conjugates was comparable to that observed on metallic carbon nanotubes and Pt nanoelectrodes. The DNA-peptide nanowires exhibited strong non-linear I–V behavior with a thousand-fold higher current as compared with bare dsDNA. The results of this work indicate that cationic peptides self-assembled on dsDNA can be used to maintain and possibly control the structure of DNA when deposited on a surface. Interestingly, this route to protect and stabilize fragile DNA molecules in a hostile environment is widely used in nature, e.g., in viruses such as M13 bacteriophage, where a DNA molecule is encased in a robust self-assembled protein tube. Our results suggest that this approach can be mimicked to stabilize and control the conductivity of long DNA stretches for applications in biosensors and molecular electronics.
